# Plasmacytoid Dendritic Cells and Cancer Immunotherapy

**DOI:** 10.3390/cells11020222

**Published:** 2022-01-11

**Authors:** Chunmei Fu, Li Zhou, Qing-Sheng Mi, Aimin Jiang

**Affiliations:** 1 Center for Cutaneous Biology and Immunology, Department of Dermatology, Henry Ford Health System, Detroit, MI 48202, USA; cfu1@hfhs.org (C.F.); lzhou1@hfhs.org (L.Z.); qmi1@hfhs.org (Q.-S.M.); 2 Immunology Program, Henry Ford Cancer Institute, Henry Ford Health System, Detroit, MI 48202, USA

**Keywords:** plasmacytoid dendritic cells, DC vaccines, exosomes, anti-tumor CD8 T cell immunity

## Abstract

Despite largely disappointing clinical trials of dendritic cell (DC)-based vaccines, recent studies have shown that DC-mediated cross-priming plays a critical role in generating anti-tumor CD8 T cell immunity and regulating anti-tumor efficacy of immunotherapies. These new findings thus support further development and refinement of DC-based vaccines as mono-immunotherapy or combinational immunotherapies. One exciting development is recent clinical studies with naturally circulating DCs including plasmacytoid DCs (pDCs). pDC vaccines were particularly intriguing, as pDCs are generally presumed to play a negative role in regulating T cell responses in tumors. Similarly, DC-derived exosomes (DCexos) have been heralded as cell-free therapeutic cancer vaccines that are potentially superior to DC vaccines in overcoming tumor-mediated immunosuppression, although DCexo clinical trials have not led to expected clinical outcomes. Using a pDC-targeted vaccine model, we have recently reported that pDCs required type 1 conventional DCs (cDC1s) for optimal cross-priming by transferring antigens through pDC-derived exosomes (pDCexos), which also cross-prime CD8 T cells in a bystander cDC-dependent manner. Thus, pDCexos could combine the advantages of both cDC1s and pDCs as cancer vaccines to achieve better anti-tumor efficacy. In this review, we will focus on the pDC-based cancer vaccines and discuss potential clinical application of pDCexos in cancer immunotherapy.

## 1. Introduction

As the sentinel of the immune system, DCs play a critical role in mediating both innate and adaptive immune responses [1]. DCs have the unique ability to initiate all adaptive immune responses to activate (prime) naive T cells, and have been recognized as the most potent “professional” antigen presenting cells (APCs) [2,3]. DCs comprise of heterogeneous populations including conventional/classical DCs (cDCs), plasmacytoid DCs (pDCs), monocyte-derived DCs (MoDCs) and Langerhans cells (LCs) that differ in their development, phenotype, localization, and functional specialization [4,5,6,7,8]. While cDCs and pDCs originate from progenitors called common myeloid progenitors (CMPs), MoDCs and LCs arise from the common monocyte progenitors (cMoPs) [9].

Developing from CMPs, macrophage/DC progenitors (MDPs) give rise to a population referred to as the common DC progenitors (CDPs), which in turn differentiate into two major DC subsets: cDCs and pDCs [4,6,7,8,10,11]. cDCs can be further divided into two major subtypes, currently described as cDC1s and cDC2s (type 2 conventional DCs) that differ in their function, phenotypes, and transcriptional factor dependency. cDC1s depend on interferon regulatory factor 8 (IRF8) and basic leucine zipper transcriptional factor ATF-like 3 (Batf3) for their development, and are identified as XCR1^hi^CD24^hi^CD26^hi^ CD11c^hi^MHCII^hi^CD11b^lo^CD172a^lo^F4/80^lo^CD64loLin^lo^ population in mice and CD141^+^ (BDCA3^+^) DCs in human [9,12,13]. In general, cDC1s are thought to be specialized in presenting exogenous, cell associated antigens onto MHCI to prime CD8 T cells, resulting in cytotoxic T lymphocyte (CTL) responses to intracellular pathogens and tumors [8]. Recent reports have shown that cDC1s played a critical role in cross-priming antigen-specific CD8 T cells in tumors to generate CD8 T cell immunity, and in determining anti-tumor efficacy of cancer immunotherapies including adoptive T cell transfer (ACT) and immune checkpoint blockade (ICB) [14,15,16,17]. Murine cDC1s include two populations: a lymphoid tissue-resident population expressing CD8a, and a non-lymphoid tissue CD103^+^ population that’s in the periphery [12]. On the other hand, cDC2s depend on interferon regulatory factor 4 (IRF4) and zinc finger E-box-binding homeobox 2 (ZEB2) for their development, and comprise of heterogeneous populations that are most efficient in priming CD4 T cells after presenting soluble antigens on MHC class II (MHCII), thus regulating immune responses against parasites, extracellular pathogens and allergens [8,18]. However, it should be noted that cDC2s could also cross-present antigens [19,20,21] and play a critical role for regulating anti-tumor CD4 and CD8 T cell responses [6,22,23,24]. Murine cDC2s are identified by their expression of CD11b and signal regulatory protein alpha (SIRPa; CD172a), and their human counterparts express CD1c (BDCA1) [9]. cDC2s are even more heterogeneous than cDC1s, and recent advance in single-cell RNA (scRNA) sequencing led to the identification of a number of cDC2 subsets [22,25,26]. Transcriptome analysis suggests that cDC2s contain at least two subsets that are ontogenetically conserved between mice and humans but with distinct functions: the T-bet-expressing cDC2A subset that are anti-inflammation, and the pro-inflammatory cDC2B population that express RORγt [27].

Besides cDC1s and cDC2s, CDPs can also give rise to at least part of the pDCs. pDCs are heterogeneous cells best known for their production of large amount of type I interferons (IFN-Is) [28,29,30]. Murine pDCs are distinguished from cDCs by their expression of Siglec-H, B220, Ly6c, PDCA1 (CD317) and intermediate CD11c, and human pDCs express CD303 (BDCA2), CD304 (BDCA4), CD123 (IL-3R), CD45RA and HLA-DR but not CD11c [29,31,32]. IRF-8 and TCF- 4 (also known as E2-2) are the main transcription factors for the development, functional specification, and maintenance of pDCs [33,34,35,36]. Expression of DNA-binding protein inhibitor ID-2, which prevents the activity of pDC transcription factor TCF-4, has to be suppressed for pDCs to be developed from CDPs. The readers are referred to several recent excellent reviews for more details on the development from CMPs to human and murine pDCs, including key transcription factors, phenotypic markers and functions that define these pDCs [8,29,31,32,37]. Besides CMP (common myeloid progenitors), in vivo transfer experiments have shown that pDCs can be generated from both CMP and CLP (common lymphoid progenitors), but are derived mainly from myeloid progenitors [38]. However, pDCs developed from CMP and CLP exhibit different function regarding type I IFN production and T cell priming [39,40]. Furthermore, murine pDCs derived from CLP showed evidence of past expression of recombination activation gene 1 (Rag1) and D-J rearrangements in IgH [40]. While previous studies suggest they are lymphoid transcriptional program transiently expressed in the pDC lineage based on their detection in pDCs developed from CMP and CLP [41,42], more recent study indicates that RAG1 expression and IgH rearrangement are mostly observed in pDCs from lymphoid progenitors [40], suggesting two distinct developmental pathways for pDC generation.

Recent single-cell RNA sequencing analysis further confirmed that pDCs can be originated from IL-7R^+^ly6D^+^ lymphoid progenitors, referred to as pre-pDCs [43,44,45]. In the first study, Rodrigues et al. have shown that while the IL-7R^+^ lymphoid precursors give rise to both pDCs and B cells, a specific subset of SiglecH^+^Ly6D^+^ cells would only differentiate into pDCs when cultured with Flt3L [43]. Similar results with the Ly6D^+^ population have been reported by another group [45], and a similar population of IL-7R^+^ progenitors was identified in humans that could differentiate into both pDCs and B cells [44]. Rodrigues et al. have further shown that only lymphoid-derived mature pDCs exhibit the capacity to process and present antigens like cDCs, although both myeloid- and lymphoid-derived mature pDCs are capable of secreting IFN-Is [43]. Further analysis also indicated that IL-7R^+^ lymphoid progenitors contribute to the majority of murine mature BM and splenic pDCs in vitro and in vivo [43], thus challenging the previous notion that pDCs are mainly derived from myeloid progenitors (see above). Taken together, these new data seem to suggest that generation of pDCs is likely regulated by cell fate decision between pDCs versus cDCs and between pDCs versus B cells. Future studies are warranted to determine the relative contribution of the two pathways to pDC generation under steady state as well as under inflammation or infection settings.

## 2. pDCs, Cross-Priming and Anti-Tumor CD8 T Cell Immunity

Cross-priming, which DCs activate (prime) CD8 T cells following presentation of exogenous antigens onto their MHCI, plays a major role in generating CD8 T cell immunity against viruses and tumors as well as mediating immune tolerance (cross-tolerance) [46,47,48,49]. In contrast to the critical role cDC1s played in cross-priming antigen-specific CD8 T cells including in tumors [14,15,16,17], whether and how pDCs function in cross-priming have remained poorly understood. In fact, whether pDCs are involved in cross-priming in vivo remains controversial [47,50,51,52], despite both murine and human pDCs having been shown to be able to cross-present antigens in vitro [21,53,54,55,56]. While multiple reports have shown that pDCs are involved in cross-priming in vivo [57,58,59,60], other studies suggested that pDCs did not [61,62,63,64,65]. The efforts to delineate the involvement of pDCs in cross-priming in vivo were further complicated by the fact that pDCs could activate and recruit B cells, cDCs, NK cells, and T cells to regulate CD8 T cell priming indirectly through their production of IFN-I [66]. For example, activated pDCs induced anti-tumor CD8 T cell responses after systemic RNA delivery, however whether this response is a result of antigen presentation by pDCs or is due to IFN-I-mediated activation of cDCs remains unclear [67].

Further complicating this issue, recent studies have shown that isolated population of pDCs used in functional studies often contain transitional pDCs that are related to both pDCs and cDCs. From human blood, BM and tonsil, a subset of CD2^+^CD5^+^CD81^+^ DCs that express multiple pDC markers (CD123, CD303, CD304) were identified, and they produced IL-12 instead of IFN-Is to prime T and B cells [68,69,70]. Furthermore, an overlapping human DC subset with pDC features has been identified by scRNA sequencing [25,71,72]. These transitional pDCs are functionally distinct from canonical pDCs: they exhibit the phenotype and functionality of cDCs in priming T and B cells, fail to produce IFN-I upon TLR7 and TLR9 stimulation, but express some pDC markers and transcription factors and require TCF4 for development. Further analysis suggested that these cells are heterogeneous and include ‘‘cDC-like’’ pre-DC cells or AXL^+^ SIGLEC6^+^ DCs (AS-DCs, or AXL^+^ DCs) [25,71,72]. These DCs represent intermediate myeloid DC populations with a mixture of pDC and cDC characteristics, raising the question that cDC function including IL-12 production and antigen presentation for T/B cell priming observed for pDCs could be due to the contaminated pre-DCs/AS-DCs. However, a recent study by Alculumbre et al. has shown that canonical pDCs activated in vitro gave rise to distinct populations with either IFN-producing or antigen-presenting functions [73]. The differentiation of PD-L1^-^CD80^+^ pDCs into cDC-like cells (antigen presentation and T cell activation) could not be explained by contamination of these transitional pDCs such as Axl^+^ DCs and likely represented an intrinsic property of activated pDCs, consistent with the notion that activated pDCs acquire the capacity to present antigens and prime T cells [57].

The roles of pDCs in cancer are similarly complex (Table 1). Poor prognosis in multiple cancers such as head and neck, melanoma, ovarian, and breast cancers have been correlated with enrichment of pDCs in tumors [29,30,74,75,76], supporting the notion that pDCs play a suppressive function in these tumors (Table 1). Indeed, pDCs have been shown to induce an inducible T cell costimulator ligand (ICOSL)-dependent expansion of regulatory T cells (Tregs) [77], and tumor-associated pDCs could activate Foxp3^+^ Tregs through indoleamine 2,3-dioxygenase (IDO) [78]. On the other hand, tumor-infiltrated pDCs have been associated with survival in human colon cancer [79], suggesting a positive role in regulating anti-tumor immunity (Table 1). Furthermore, activation of pDCs has been shown to induce anti-tumor immunogenic responses and several pDC clinical trials have shown promising clinical benefits in human cancers, suggesting that pDCs could be employed to induce anti-tumor immunity [64,80,81,82,83,84,85,86] (Table 1). As we have discussed above, it remains unclear whether pDCs function by cross-prime CD8 T cells directly or by activating cDCs and other immune cells indirectly through their production of cytokines including IFN-Is [87,88]. For instance, CpG-activated pDCs induce tumor regression by recruiting NK cells through IFN-Is to enhance tumor antigen-specific CD8 T cell cross-priming [80]. Despite the uncertainty of how pDCs function to induce anti-tumor immunity, however, pDCs likely play some roles in regulating cross-priming to generate CD8 T cell immunity, as increasing evidences have shown that cooperation of pDCs and cDCs are required to induce optimal cross-priming and CD8 T cell immunity under different settings [80,89,90,91,92,93]. Using an in vivo pDC-targeted vaccine model, we have recently shown that antigen-targeted pDCs required bystander cDCs to cross-prime antigen-specific CD8 T cells [94], which might provide one potential explanation for the conflicting results regarding pDCs’ role in cross-priming in vivo. It should be noted, however, that both human and murine pDCs have been shown to be capable of directly killing tumor cells through granzyme B- and/or tumor necrosis factor-related apoptosis-inducing ligand (TRAIL)-dependent mechanisms [95,96,97,98] (Table 1).

## 3. Current pDC-Based Cancer Vaccine Clinical Trials

Three clinical trials using pDCs as cancer vaccines including two phase I and one phase II clinical trials have been reported [82,85,86] (see Table 2). Several clinical trials using pDCs or combination of pDCs and cDCs are ongoing and have not reported their findings: a phase I and II clinical trial NCT-02574377 for melanoma patients using pDCs, cDCs and combination of both pDCs and cDCs; a phase IIa clinical trial for prostate cancer using pDCs, cDCs and combination of both pDCs and cDCs; a phase III clinical trial NCT-02993315 on melanoma patients with combination treatment of both pDCs and cDCs [99], and a phase I and II clinical trial NCT-03970746 which will determine the safety, tolerability, immunogenicity and clinical activity of a pDC cell line-based cancer vaccine, either with or without anti-PD-1 treatment in patients with non-small-cell lung cancer.

### 3.1. Phase I Clinical Trials for pDC Cancer Vaccines

In the first clinical trial using naturally occurring plasmacytoid dendritic cells (pDC) as cancer vaccines, 15 HLA-A2^+^ patients with metastatic melanoma were enrolled in a phase I clinical trial to determine the safety and dosage of pDC vaccine using clinical grade mature pDCs [82]. pDCs were isolated from apheresis products directly with the Miltenyi Biotec immunomagnetic CliniMACS isolation system under Good Manufacturing Practices (GMP) condition. This procedure generated pDCs with 75% purity that’s clinically applicable and a yield of 13–33 × 10^6^. Isolated pDCs were then cultured overnight with recombinant human interleukin-3 (rhIL-3), and activated by Fruhsommer-meningoencephalitis (FSME, tick-borne encephalitis) and loaded with three tumor-associated antigens (TAAs) including gp100_154–162_, gp100_280–288_ and tyrosinase-derived peptide tyrosinase_369–377_ (TYR_369–377_). Using this procedure, obtained mature pDCs were more than 50% viable; exhibited high expression of MHC class I and II, CD80, CD86, CD83, and CCR7, and secreted IFN-a. Fifteen patients were vaccinated with increasing pDC numbers up to 3 × 10^6^ pDCs for each vaccination, with three vaccinations at day 1, 14 and 21.

The pDC vaccines were well-tolerated and only grade 1–2 adverse effects were observed. Upon pDC vaccination, the authors observed: (1) the migration of injected pDCs as detected by in vivo imaging, (2) a type I IFN signature after each vaccination, (3) CD4 T cell proliferation and antibody production to the FSME antigens, and (4) induction of tumor antigen-specific CD8 T cell responses. Taken together, these data suggest that vaccination with naturally occurring pDCs is feasible and well-tolerated in advanced melanoma patients, and induces favorable immune responses. Although this phase I clinical trial with small number of patients was not designed for examining clinical outcome, pDC vaccination did lead to clinical benefits to several patients. Two patients exhibited durable stable disease and thus received two additional maintenance treatments consisting of three biweekly pDC vaccinations; one patient developed a mixed response was observed in one patient who exhibited lung metastasis regression but progressed on a nodal metastasis; and one patient had complete remission following surgery and subsequent pDC maintenance treatment.

In addition, to determine whether pDC vaccination affects clinical outcome, the authors retrospectively examined the clinical outcome of control patients who received a first-line treatment of dacarbazine (DTIC) chemotherapy and pDC-vaccinated patients. The pDC vaccine group exhibited a slightly increase in median PFS compared to the control group (4.0 versus 2.1 months, not significant). However, pDC vaccine group showed a significant improvement on their median OS (overall survival) over control patients: 22.0 (95% confidence interval (CI), 1.8–42.2) compared to 7.6 months (95% CI, 5.8–9.4). Perhaps more importantly, 7 out of 15 patients were alive 2 years after pDC vaccination, whereas 6 of 72 patients from the control group were alive. Thus, while the phase I clinical trial was designed for safety and feasibility, the significant difference in OS in the retrospective study comparing pDC vaccine group and standard chemotherapy group called for future phase II clinical trial to assess the potential of natural occurring pDCs as cancer vaccines.

Recently, Charles et al. reported the findings from the GeniusVac-Mel4 phase I clinical trial (Table 2), which determined the feasibility, safety, and tolerance of multiple subcutaneous immunizations of pDC vaccines in patients with metastatic melanoma [86]. The most exciting part of this clinical trial is that pDCs were obtained from a human pDC cell line instead of autologous pDCs from patients. This proprietary allogeneic plasmacytoid dendritic cell line is derived from malignant leukemic cells of a pDC leukemia patient, and have been shown to induce antigen-specific T cell responses in pre-clinical studies [100,101,102]. Two clinical batches of pDC cell line were generated from the primary cell bank, and then were amplified in CellSTACK Chambers. The Drug Product (DP) was composed of four cellular preparations, each of them loaded with one of four melanoma antigens separately: MART1_26–35L_ (ELAGIGILTV), gp100_209–217_ (FLWGPRALV), TYR_369–377_ (IMDQVPFSV), and MAGEA3_271–279_ (YMDGTMSQV), and then irradiated to prevent further proliferation of pDCs in the patients. Nine recruited patients were split into three groups in three doses (4, 20 or 60 × 10^6^ cells per vaccination) and received three weekly immunizations of the DP (irradiated pDCs). The pDC vaccine was safe and well tolerated with no serious vaccine-induced side effects for three weekly immunizations of up to 60 × 10^6^ cells [86]. Blood samples at multiple time points (Week 1, 2, 4, 8, 12, 16, 24, 36, and 48) were examined for immuno-monitoring. Interestingly, there was no allogeneic responses to the vaccines, consistent with previous observation on allogenic DC vaccines [103]. Antigen-specific T cell responses against the four epitopes were evaluated using peripheral blood with no in vitro activation or expansion. As the detection threshold of the frequency of antigen-specific T cells was about 0.01% of the total CD8 T cells, no MAGEA3-specific and gp100-specific CD8 T cells were detected, and TYR-specific T cells were detected in one out of nine patients. However, MART1-specific CD8 T cells, which have been shown to be detectable more easily, were detected in six of nine patients. Two of the six patients exhibited a significant increase of MART1-specific CD8 T cells compared to baseline. More interestingly, these MART1-specific CD8 T cells exhibited memory phenotype at two or more time points examined.

For clinical outcome, two patients from 60 × 10^6^ cell group had stable disease throughout the study. Four patients were alive at 48 weeks and two patients required no other treatment, suggesting that GeniusVac-Mel4 pDC vaccine might have slowed tumor progression. Two of the four patients also had concomitantly worsened vitiligoid lesions indicative of an anti-melanocyte immune response, suggesting that the pDC vaccines might contribute to their favorable clinical outcome. Taken together, these data suggested that the GeniusVac-Mel4 pDC vaccine induced antigen-specific T cell responses indicative of clinical responses.

The authors also carried out detailed analysis of the metastasis, and observed that tumor antigen-specific T cells were excluded from the tumor bed following being recruited into the metastasis from the blood, and the PD-1/PD-L1 axis might play a negative role in suppressing anti-tumor immunity by antigen-specific CD8 T cells primed or re-stimulated by pDC vaccines. Based on these findings, the authors examined whether pDC vaccine and PD-1 blockade exhibit synergy in priming tumor antigen-specific CD8 T cells in an ex vivo study of PBMC from healthy volunteers and melanoma patients. The synergistic effects of combination treatment of pDC vaccine and PD-1 blockade (Pembrolizumab) was examined by determining the induction of MART1-specific CD8 T cells of 12 melanoma patients after co-culture. Indeed, enhanced induction of MART1-specific CD8 T cells was observed by combination treatment with MART1-loaded pDC vaccine and PD-1 blockade compared to treatment with pDC vaccine alone without PD-1 blockade. Thus, pDC vaccine may synergize with ICI (Immune checkpoint inhibition) to further augment anti-tumor immunity to improve pDC vaccine efficacy.

As the cell line expresses HLA-A2.1, the study treatment was restricted to MHCI matched (HLA-A2.1) patients. However, the pDC cell line also expresses other HLA molecules, and could potentially be engineered to express other HLA molecules to treat patients expressing these HLA molecules. As the use of pDC cell line could supply pDCs without limitation, is compatible to manufacture standard and has no burden on cancer patients, future studies are warrantied to determine whether pDC cell lines could be employed as the alternative to naturally circulating DCs or in vitro cultured DCs used in current clinical trials.

### 3.2. Phase II Clinical Trial for pDC Cancer Vaccines

Westdorp et al. have recently reported on a phase IIa clinical trial for castration-resistant prostate cancer (CRPC) with naturally occurring pDCs and cDC2s [85], which is one of the clinical trials carried out by the same group using naturally occurring pDCs, cDC2s or a combination of both pDCs and cDC2s [99]. CD1c^+^ cDC2s and pDCs were obtained from apheresis products directly with the Miltenyi Biotec CliniMACS isolation system under GMP condition. cDC2s and pDCs were then stimulated with protamine/mRNA and loaded with HLA-A2.1 restricted TAA epitopes: MAGE-C2_336–344_ (ALKDVEERV), NY-ESO-1_157–165_ (SLLMWITQC) together with PepTivators for both MUC1 and NY-ESO-1 that cover the complete epitopes for both MHCI and II with overlapping long peptides. The obtained cDC2s and pDCs were more than 50% viability, >50% purity, >50% CD80^+^ on pDCs and >50% CD83^+^ on mDCs. These cells were frozen and stored at <−80 °C for up to 2 years and thawed on the day of vaccination. A total of 21 chemo-naive CRPC patients were assigned in a 1:1:1 ratio randomly to receive maximally nine vaccinations with mature cDC2s, pDCs or both pDCs and cDC2s.

Immunological response after DC vaccination was used as primary endpoint, by monitoring peripheral blood and T cell cultures of biopsies following delayed-type hypersensitivity-skin (DTH) tests after vaccination. Safety, feasibility, radiological progression-free survival (rPFS) and overall survival were the main secondary endpoints. All DC vaccinations were well tolerated and only grade 1–2 toxicity was observed. DTH tests were carried out after each cycle of 3 DC immunizations to study MAGE-C2-, MUC1- and NY-ESO-1-specific T cell responses. NY-ESO-1-specific CD8 T cells were detected in 15 out of 21 patients. MUC1- and MAGE-C2-specific CD8 T cells were detected in 5 and 12 out of 21 patients, respectively. IFN-γ production was detected in 8 of 21 patients when skin-test infiltrating lymphocytes (SKILs) were examined for induction of T helper 1 (Th1) cytokines. No significant differences in TAA-specific responses were observed among patients in pDCs, mDC2s or the combination groups. In peripheral blood, NY-ESO-1-specific CD8 T cells were detected in 7 of 21 patients prior to DC vaccinations, and no detection of MAGE-C2- or MUC1-specific CD8 T cells in any patient. Vaccine antigen-specific T cells were detected in 12 of 21 patients after vaccination: MUC-1, MAGE-C2- and NY-ESO-1-specific T cells were detected in 2, 4 and 10 out of 21 patients, respectively. Antigen-specific T cells against multiple TAAs were detected in 4 out of 21 patients.

For clinical outcome, 12 patients achieved stable disease that persisted > 6 months, and a partial radiological response was observed in 1 patient. Median rPFS for all patients was 9.5 months. No significant difference was observed among the three treatment groups: 12.0 months rPFS for mDC group, 10.7 months in the pDC group and 4.2 months for the combination group. The presence of functional T cells specific for vaccine antigens correlated with longer rPFS. Skin biopsies of patients with radiological non-progressive disease exhibited higher detection frequency (5/13 patients) of tetramer/dextramer-positive (dm^+^) and IFN-γ-producing (IFN-γ+) antigen specific T cells compared to skin biopsies of patients with progressive disease (0/8 patients). In patients with functional (dm^+^ and IFN-γ^+^) T cells specific for vaccine antigens after DC vaccination, median rPFS was 18.8 months vs. 5.1 months in dm^−^ patients or patients without IFN-γ-producing antigen-specific T cells. Patients with functional (dm^+^ and IFN-γ^+^) T cells specific to vaccine antigens also exhibited longer prostate-specific antigen doubling time (PSAdt) at 6 months compared to IFN-γ^−^ or dm^−^ patients (mean PSAdt 12.9 vs. 8.6 months). In 2 of 21 patients PSA level was decreased, and one of these patients showed a partial radiological response and > 99% PSA-decrease. Median OS (overall survival) was not reached. However, patients with functional (dm^+^ and IFN-γ^+^) antigen-specific T cells have longer OS than patients without these cells (dm^−^ or IFN-γ^−^ patients), suggesting that vaccine-induced functional antigen-specific T cells might have an OS benefit.

TAA expression was examined on prostate biopsies or radical prostatectomy tissue to determine whether DC vaccination regulated TAA expression of the primary tumors. Interestingly, patients with T cells specific to TAA whose tumors expressed (dm^+^ and tumor^+^) had a median rPFS of 10.7 months, whereas patients without T cells specific to their tumor TAA (dm^+/−^ and tumor^−^) had a 5.2-month median rPFS. However, the difference was not statistically significant, likely due to the small sample size. In patients with disease progression, two patients showed loss of tumor MUC1 expression, and one of the patients had MUC-1-specific T cells, suggesting that tumors might evade DC vaccine-induced anti-tumor immunity by down-regulating TAAs.

Taken together, the phase IIa clinical trial using naturally circulating DCs (pDCs and/or cDC2s) has demonstrated that vaccinations with naturally circulating cDC2s and/or pDCs are safe and well-tolerated in CRPC patients. Vaccination with these DCs induced T cell responses specific to vaccine antigens in a majority of patients. Patients with functional vaccine antigen-specific T cells exhibited significantly higher median rPFS and likely OS benefit compared to patients without these cells, suggesting vaccination with naturally circulating DCs might boost long-term cancer control especially in combination with other cancer therapies.

## 4. Would Plasmacytoid DC-Derived Exosomes Be Used as Cancer Vaccines?

DCs process exogenous antigens in endosomal compartments including multivesicular endosomes which can release small inert vesicles 30–150 nm in diameter called exosomes (DCexos) following fusing with plasma membrane [104,105,106,107]. DCexos contain proteins, metabolites and nucleic acids including miRNAs (microRNAs), and play significant roles in intercellular communications and material transfer of their cargo [104,107]. DCexos additionally express complexes of MHC class I/II-antigenic epitopes and co-stimulatory molecules, making them capable of priming antigen-specific CD8 T cells [108,109,110,111]. Due to their resistance to tumor-mediated suppression, biostability and bioavailability, DCexos have garnered great interest as cell-free therapeutic agents [104,105,107]. In one study, Zitvogel et al. have shown two decades ago that vaccination with DCexos (from BM-derived DCs) led to better anti-tumor efficacy than vaccination with DCs [112], thus providing support of the development of DCexo vaccines for cancer treatment. However, current DCexo clinical trials using peptide-pulsed autologous DCexos have only shown limited clinical benefits and failed to induce antigen-specific T cell responses [113,114,115]. Whether pDCs generate exosomes and how these exosomes function have not been examined, although exosomes have been shown to regulate the functions of pDCs [116,117,118]. Therefore pDC-derived exosomes (pDCexos) have not been explored as cancer vaccines even as multiple clinical trials using pDCs have shown promising data [82,85,86].

Although these pDC clinical trials have clearly shown that vaccination with pDCs could lead to induction of anti-tumor CD8 T cell immunity [82,85,86] despite their presumed tolerogenic role in tumors in multiple tumors [29,30,74,75,76], how pDCs achieve these opposite function through cross-priming remains poorly understood [87,88]. In fact, whether pDCs are involved in cross-priming in vivo remains controversial [47,50,51,52]. To determine whether and how pDCs cross-prime antigen-specific CD8 T cells in vivo, we decided to examine vaccine-induced CD8 T cell responses with a pDC-targeted vaccine model [94]. Anne Krug’s group has previously shown that antibodies targeting pDC specific receptors Siglec-H and Bst2 delivered antigens to pDCs but not cDCs in vivo [119,120]. Using an in vivo pDC-targeted vaccine model with these antibodies, we have reported that while pDC-targeted vaccination with adjuvant resulted in strong cross-priming and CD8 T cell immunity, antigen-targeted pDCs required non-targeted cDCs to achieve cross-priming in vivo [94]. More interestingly, the non-targeted cDCs also expressed the MHCI-antigen complexes (pMHCI) on their surface, together with the fact that pDC-targeted antibodies were not detected in the non-targeted cDCs, indicating that antigen-targeted cross-presenting pDCs likely transfer antigens to cDCs [94]. Indeed, non-targeted cDCs but not pDCs were able to cross-prime naïve OVA-specific CD8 T cells ex vivo, further confirming that cross-presenting pDCs co-operate with non-targeted bystander cDCs to cross-prime antigen-specific CD8 T cells in vivo. We further confirmed this conclusion with an in vitro co-culture system where we limited antigen access to pDCs. Similar to our ex vivo results, cross-presenting pDCs primed naive antigen-specific CD8 T cells only in the presence of antigen-naïve bystander cDCs by transferring antigens to bystander cDCs, suggesting that cross-presenting pDCs require bystander cDCs for cross-priming in vivo and in vitro by transferring antigens to bystander cDCs [94]. As cDCs could be further divided into cDC1s and cDC2s, we asked whether cDC1s and cDC2s had different roles in pDC-mediated cross-priming. Using Batf3^−/−^ mice that lack of both CD8^+^ and CD103^+^ cDC1s, we were able to show that cDC1s were required for pDC-mediated cross-priming upon in vivo pDC-targeted vaccination. Surprisingly, cDC1s and cDC2s exhibited similar efficiency in acquiring antigens from cross-presenting pDCs, thus suggesting that cDC1s played a critical role in pDC-mediated cross-priming after antigen presentation. As Batf3-dependent cDC1s have been suggested as the DC subset specialized in cross-presenting exogenous antigens including tumor antigens to prime CD8 T cells [10,11,13], our data raise the interesting scenario that pDCs and cDC1s might induce cross-priming synergistically.

What are the underlying mechanisms of how cross-presenting pDCs transfer antigens to bystander cDCs? We further demonstrated that antigen transfer to bystander cDCs from pDCs was mediated by pDCexos. Importantly, pDCexos induced cross-priming of antigen-specific CD8 T cells only in the presence of bystander cDCs, similar to pDCs, suggesting that pDCs achieve cross-priming by transferring antigens to cDCs through pDCexos [94]. Given the critical role of cDC1s in pDC-mediated cross-priming, our data additionally suggested that pDCexos might similarly require cDC1s to achieve optimal cross-priming [94].

## 5. Conclusions and Future Perspectives

Our findings that cross-presenting pDCs co-operated with cDC1s to achieve optimal cross-priming [94] add another line of evidence that cooperation of pDCs and cDCs might be necessary to induce optimal cross-priming and to generate durable CD8 T cell immunity [80,89,90,91,92,93]. Interestingly, a recent study has shown that while BDCA1^+^ cDC2s are better at inducing antigen-specific CD8 T cell responses, pDCs are more efficient in activating NK cells [121]. As all subsets of DCs could contribute to anti-tumor immune responses, it is likely that the most efficient DC vaccines will have multiple DC subsets to take advantage of their complementary functions and crosstalk between innate and immune cells. Thus, incorporating multiple subsets of DCs might be a feasible approach to improve DC vaccine efficacy. As no significant difference in rPFS was observed with combined vaccine using both cDC2s and pDCs in the phase IIa CRPC clinical trial [85], future phase III clinical trials will need to assess the efficacy of combined vaccines with both pDCs and cDCs.

Our identification of the previously unreported pDCexos not only adds an exciting new type of DCexos to current repertoire, but also offer pDCexos as a new type of DC-based vaccines that would potentially combine the advantages of DCexo and pDC vaccines. As inert vesicles, pDCexos would be more resistant to tumor-mediated immunosuppression and more biostable compared to pDCs, and could be obtained from multiple well-characterized human pDC cell lines [86,122,123,124] without demanding procedures on vaccine patients. Further characterization of these newly identified pDCexos will be required to determine their potential application as cancer vaccines. In addition, future studies are warrantied to investigate exosomes from other naturally circulating DCs as well as DCs differentiated in vitro under different conditions to determine their potential in cancer vaccines [82,125,126,127,128,129,130].

## Figures and Tables

**Table 1:** Opposite functions of pDCs in tumors.

Function in Tumors	Phenotypes and Mechanisms	References
Negative role in anti-tumor immune responses	Accumulation of pDCs correlated with poor diagnosis in multiple tumors. Potential mechanisms include induction of regulatory T cells through ICOSL- or IDO-dependent pathways.	[29,30,74,75,76,77,78]
Positive role in anti-tumor immunity	Tumor-infiltrated pDCs correlate with survival in human colon cancer, and activation pDCs lead to enhance anti-tumor immunity. Possible mechanisms include: IFN-I-dependent enhancement of function of NK cells and T cells, as well as cross-priming by cDCs; enhanced direct cross-priming. However, the exact contributions of IFN-I versus pDC-mediated cross-priming remain poorly understood.	[64,79,80,81,82,83,84,85,86]
Tumoricidal activity	Activated pDCs directly kill tumor cells through TRAIL- and Granzyme B-dependent mechanisms leading to tumor regression.	[95,96,97,98]

**Table 2:** Current pDC-based clinical trials.

Cancer Type	Phase	pDC Used	Doses	Patients	Toxicity	Clinical Outcomes
gp100- expressing distant metastatic melanoma	I	Isolated naturally occurring mature pDCs were loaded with gp100_154–162_, gp100_280–288_ and tyrosinase-derived peptide tyrosinase_369–377_.	Three intranodal injections every 2 weeks. Two maintenance cycles consisting of 3 biweekly vaccinations if no disease progression	Fifteen HLA-A2^+^ patients with distant metastatic melanoma	Only grade 1–2 toxicity	Generation of CD8 T cell responses specific to tumor antigens; two patients showed durable stable disease and were eligible for 2 additional cycles consisting of 3 pDC vaccinations. One patient with a mixed response [82].
Castration-resistant prostate cancer	IIa	Blood-derived pDCs, CD1a+ cDC2s or a combination of pDCs and cDC2s, loaded with NY-ESO-1_157–165_, MAGE-C2_336– 344_ and NY-ESO-1 and MUC1 PepTivators (overlapping long peptides that cover the complete protein).	Maximal 9 times	21 (21 HLA-A2^+^ patients with confirm adenocarcinoma of the prostate, 7 for each treatment)	Grade 1–2 toxicity	A partial radiological response was observed in 1 patient; 12 patients (57%) with stable disease > 6 months. No significant difference among the three treatment arms (cDCs, pDCs and cDCs + pDCs) [85].
Stage IIIC or IV confirmed unresectable metastatic melanoma	Ib	pDCs from a cell line loaded with one of three melanoma antigens separately: MART1_26–35L_, MAGEA3_271–279_, gp100_209–217_ and TYR_369–377_.	3 weekly injections	9 HLA-A2^+^ stage IIIC or IV patients with confirmed unresectable meta- static melanoma	Grade 1–3 toxicity	Three weekly injections of up to 60 × 10^6^ cells were safe and well tolerated. Two patients from the highest dose group (60 × 10^6^ cells) displayed a stable disease [86].
